# Monitoring translation in all reading frames downstream of weak stop codons provides mechanistic insights into the impact of nucleotide and cellular contexts

**DOI:** 10.1093/nar/gkac1180

**Published:** 2022-12-19

**Authors:** Gary Loughran, Xiang Li, Sinead O’Loughlin, John F Atkins, Pavel V Baranov

**Affiliations:** School of Biochemistry and Cell Biology, University College Cork, Cork, Ireland; School of Biochemistry and Cell Biology, University College Cork, Cork, Ireland; School of Biochemistry and Cell Biology, University College Cork, Cork, Ireland; School of Biochemistry and Cell Biology, University College Cork, Cork, Ireland; Department of Human Genetics, University of Utah, Salt Lake City, UT 84112, USA; School of Biochemistry and Cell Biology, University College Cork, Cork, Ireland

## Abstract

A stop codon entering the ribosome A-site is normally decoded by release factors that induce release of the polypeptide. Certain factors influence the efficiency of the termination which is in competition with elongation in either the same (readthrough) or an alternative (frameshifting) reading frame. To gain insight into the competition between these processes, we monitored translation in parallel from all three reading frames downstream of stop codons while changing the nucleotide context of termination sites or altering cellular conditions (polyamine levels). We found that P-site codon identity can have a major impact on the termination efficiency of the *OPRL1* stop signal, whereas for the *OAZ1* ORF1 stop signal, the P-site codon mainly influences the reading frame of non-terminating ribosomes. Changes to polyamine levels predominantly influence the termination efficiency of the *OAZ1* ORF1 stop signal. In contrast, increasing polyamine levels stimulate readthrough of the *OPRL1* stop signal by enhancing near-cognate decoding rather than by decreasing termination efficiency. Thus, by monitoring the four competing processes occurring at stop codons we were able to determine which is the most significantly affected upon perturbation. This approach may be useful for the interrogation of other recoding phenomena where alternative decoding processes compete with standard decoding.

## Graphical Abstract

**Graphical Abstract:**
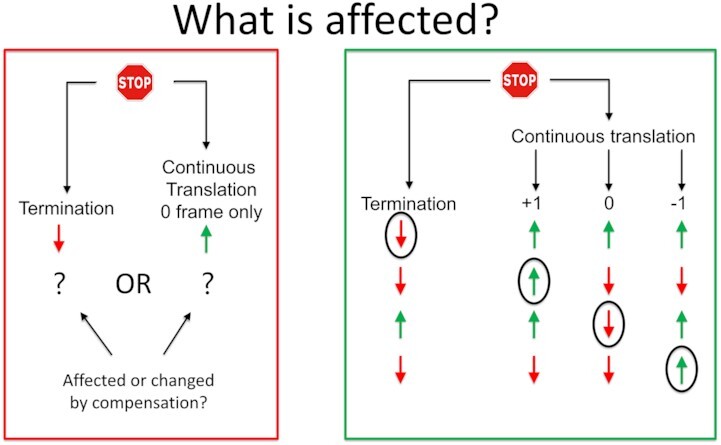
Monitoring more than two competing processes allows detection of the process most affected by a change (sequence or cellular environment) as it anticorrelates with all others by compensation.

## INTRODUCTION

Translation termination involves the hydrolytic release of the nascent polypeptide chain from the peptidyl-tRNA by protein release factors upon encountering a stop codon (UAA, UAG or UGA in the standard genetic code) in the ribosome A-site (1,2). In most eukaryotes, a single release factor (eRF1) recognizes all three stop codons. Normally the efficiency of termination is very high (>99.9%) but is influenced by *cis*-acting elements (3–16) and/or *trans*-acting factors (17–26). Reduced termination efficiency can increase the chances of near-cognate tRNA successfully competing with release factor for ribosomal A-site acceptance, resulting in continued translation in the same (zero) reading frame (readthrough). Both processes also compete with incorporation of tRNAs cognate to A-site codons in alternative frames (frameshifting). When synthesis of proteoforms with alternative C-terminal extensions increases evolutionary fitness, sequences surrounding a stop codon may evolve to reduce the efficiency of termination to increase either stop codon readthrough or frameshifting.

Stop codon readthrough is well known in decoding of viral genes, especially of RNA viruses (27–32), and is unusually common in fruit flies (33–37) and mosquitoes (38). Until relatively recently, hardly any instances of experimentally verified conserved mammalian readthrough were known (39). Advances in sequencing technologies paved the way for the advent of ribosome profiling (40) which has identified several human readthrough genes (35,41–44). An increase in the number of species with sequenced genomes has also strengthened the power of comparative genomics which led to the identification of mammalian mRNAs whose translation probably involves stop codon readthrough (9,34,45–47). Subsequent experimental analysis confirmed extended in-frame decoding beyond the annotated stop codon for several of these readthrough candidates (48–52).

In most instances of stop codon readthrough, the identity of the near-cognate tRNA, and hence the amino acid specified, is believed to be generally unimportant, with the key feature being the encoded C-terminal extension. This is in contrast to some forms of cognate decoding (or redefinition) of stop codons where the non-universal amino acids selenocysteine or pyrrolysine are specified and the identity of the amino acid encoded is of key functional significance (53).

Although the mechanism of *cis*-acting regulation of termination efficiency is still unclear, cryo-electron microscopy (EM) structures of mammalian ribosomes containing A-site-bound eRF1 revealed that eRF1 binding induces an unusual conformation in which four mRNA nucleotides—the stop codon and one additional 3′ nucleotide—occupy the A-site (54,55). This conformation allows the two nucleotides beyond the stop codon (+4 and +5) to stack with 18S rRNA bases G626 and C1698, respectively. However, some studies have revealed *cis*-acting influences on termination efficiency as much as 12 nucleotides 3′ of the stop codon (8,9), suggesting that these extended stop signals may reduce termination efficiency by base pairing with rRNA within the mRNA entrance tunnel to prevent eRF1 from ‘pulling’ the fourth base into the A-site (8,9).

Previously we identified UGA_CUAG as a mammalian readthrough-prone extended stop signal (9,45). This readthrough motif was subsequently corroborated using different approaches by several groups (8,51,56,57). Only 23 human genes contain this stop signal; however, their readthrough efficiencies vary by >10-fold, with *DUS4L* having the lowest readthrough efficiency (∼1.5%) and *OPRL1* the highest (∼17%) (46). Our initial goal was to identify readthrough stimulatory sequences within the *OPRL1* mRNA by systematically comparing it with the readthrough signal from *DUS4L—*this revealed an important role for the P-site codon. However, unlike in many previous studies of stop codon readthrough that focus on readthrough as the sole alternative to termination, we also monitored ribosomal frameshifting into both alternative frames in parallel for *OPRL1*. This allowed us to determine which of these competing processes is most affected by changes to codons in the P-site. We further extended this approach to analyse the stop codon at the first open reading frame (ORF1) of human antizyme 1 (*OAZ1*), where +1 frameshifting is the predominant alternative to termination (58). We also demonstrated the applicability of this approach for measuring the effect of cellular factors such as polyamine levels on termination.

## MATERIALS AND METHODS

### Plasmids

*OPRL1* and *DUS4L* dual luciferase expression constructs were generated by ligating the annealed oligonucleotide pairs outlined in Supplementary Table S1 into PspXI/BglII-digested pSGDlucV3.0 [Addgene 119760; (45)]. *OAZ1* dual luciferase expression constructs were generated by either one-step or two-step polymerase chain reaction (PCR) on an *OAZ1* G Block (IDT) using primer sequences outlined in Supplementary Table S1 which incorporated 5′ XhoI and 3′ BglII restriction sites. PCR amplicons were digested with XhoI/BglII and cloned into PspXI/BglII-digested pSGDlucV3.0 (Addgene 119760). All clones were verified by Sanger sequencing (Eurofins).

### Cell culture and transfections

Human embryo kidney (HEK) 293T cells (ATCC) were maintained in Dulbecco’s modified Eagle’s medium (DMEM) supplemented with 10% foetal bovine serum (FBS), 1 mM l-glutamine and antibiotics. Cells were transfected with Lipofectamine 2000 reagent (Invitrogen), using the 1 day protocol in which suspended cells are added directly to the DNA complexes in half-area 96-well plates. The following were added to each well: 25 ng of each plasmid plus 0.2 μl of Lipofectamine 2000 in 25 μl of Opti-Mem (Gibco). The transfecting DNA complexes in each well were incubated with 3 × 10^4^ cells suspended in 50 μl of DMEM + 10% FBS at 37°C in 5% CO_2_ for 20 h.

For polyamine depletion experiments, cells were split and grown overnight to ∼70% confluence, then 4 × 10^6^ cells were plated in 10 cm plates in medium supplemented with 2.5 mM α-difluoromethylornithine (DFMO; a kind gift from P. Woster via Dr Michael Howard, University of Utah). Cells were incubated for 5 days in DFMO-supplemented media at 37°C in 5% CO_2_ and then transfected using Lipofectamine 2000 reagent (Invitrogen) as described above. Suspended cells were supplemented to final concentrations of 2.5 mM DFMO, 1 mM aminoguanidine hydrochloride (Sigma) or the same plus 2 mM (final) spermidine (Sigma) before adding to transfecting DNA complexes. Luciferase activities were measured 24 h after spermidine treatment.

### Dual luciferase assay

Relative light units were measured on a Veritas Microplate Luminometer with two injectors (Turner Biosystems). Transfected cells were lysed in 15 μl of 1× passive lysis buffer (PLB: Promega), and light emission was measured following injection of 50 μl of either *Renilla* or firefly luciferase substrate (59).

Recoding efficiencies were determined by calculating relative luciferase activities (firefly/*Renilla*) from test constructs and dividing by relative luciferase activities from replicate wells of matched in-frame control constructs. Three replicate biological samples were assayed, each with four technical repeats. Statistical significance was determined using a two-tailed, homoscedastic Student's *t*-test.

## RESULTS AND DISCUSSION

### The upstream sequence has a higher contribution to the difference between *OPRL1* and *DUS4L* readthrough efficiencies than the downstream sequence

**Figure 1.**
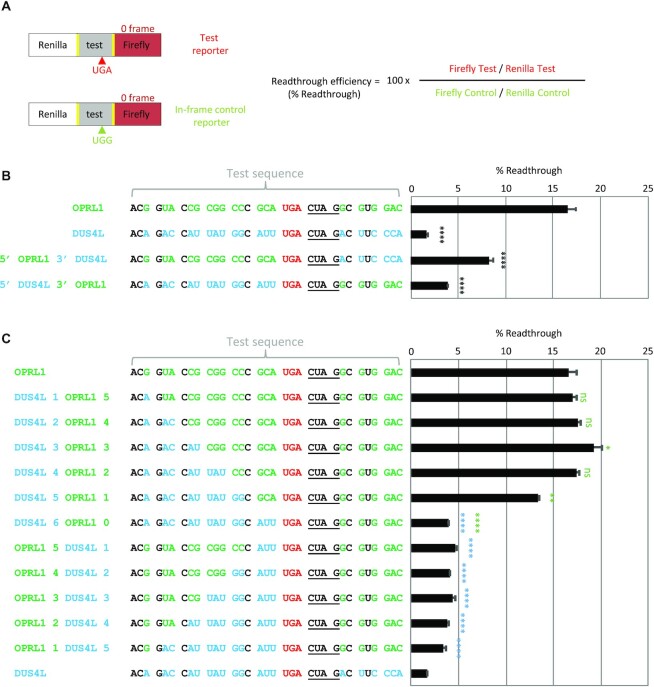
The upstream sequence has a higher contribution to the difference between *OPRL1* and *DUS4L* readthrough efficiencies than the downstream sequence. (**A**) Cartoon depicting the reporter system used in this study. Readthrough efficiencies (% Readthrough) of tested stop signals are assessed by transfecting test and in-frame control dual luciferase reporters in parallel into HEK293T cells and then calculating the ratio of relative luciferase activities from test and control transfections. (**B**) Readthrough efficiencies of the *OPRL1* and *DUS4L* stop signals. *OPRL1*-specific nucleotides are in green font, *DUS4L*-specific nucleotides are in blue font and identical nucleotide positions are in black font. The UGA stop codon is in red font and the CUAG readthrough motif is underlined. (**C**) Readthrough efficiencies of the *OPRL1* and *DUS4L* stop signals with codon replacements as indicated. Statistical significances were determined in comparison with *OPRL1* in (**B**) or with *OPRL1* (green asterisks) and *DUS4L* (blue asterisks) in (**C**). *n* = 3, ns, not significant >0.05; *P <0.05; **P <0.01: ***P <0.001; ****P <0.0001 by Student's two-tailed *t*-test.

Our approach for the measurement of recoding efficiencies takes advantage of a dual luciferase reporter system that we previously described (45) (Figure 1A). Briefly, the test sequence is flanked by tandem StopGo sequences [from foot and mouth disease virus (60)] that prevent peptide bond formation at a specific site, resulting in expression of both reporters as separate proteins. This arrangement avoids potential artefacts that can arise when the test sequence product fusion alters the individual reporter activities or their stabilities. This system has been instrumental in elucidating frameshifting and readthrough artefacts (45,61,62).

We first tested wild-type *OPRL1* and *DUS4L* readthrough cassettes in HEK293T cells with just 18 nt 5′ of the stop codon and 12 nt 3′ to reconfirm a >10-fold difference in readthrough efficiency between *OPRL1* and *DUS4L* stop signals in mammalian cells even though their UGA stop codons both have the 3′-adjacent CUAG readthrough motif (Figure 1B). There are 18 nt differences between the *OPRL1* and *DUS4L* cassettes—13 of these differences are 5′ of the stop codon and 5 are 3′ (*OPRL1-*specific nucleotides in green font and *DUS4L*-specific nucleotides in blue font in Figure 1). In order to determine the *cis*-acting signals responsible for the difference in their readthrough efficiencies, we swapped *OPRL1* and *DUS4L* 5′ and 3′ sequences. This revealed that the 3′ part is responsible for an ∼2-fold difference whereas the 5′ part is responsible for an ∼4-fold difference (Figure 1B), thus indicating that while both *cis*-acting contexts contribute to the readthrough differences, the major discriminatory signal is located 5′ of the stop codon. To determine the nature of this signal in more detail, we focused on the 5′ part hereafter.

### The identity of the P-site codon is a major factor determining readthrough efficiency

To locate the discriminatory 5′ signal, we tested readthrough efficiencies in constructs where we systematically replaced the six codons 5′ of the *OPRL1* stop codon with corresponding codons from *DUS4L*. Only when both E- and P-site codons (CCC_GCA of *OPRL1*) were replaced with corresponding *DUS4L* codons (GGC_AUU) did we observe any decrease in readthrough efficiency (Figure 1C). Replacing the *OPRL1* E-site codon (CCC) only reduced readthrough efficiency by ∼1.25-fold, whereas replacing the P-site codon (GCA) caused a further ∼3-fold decrease. Reciprocal experiments where the *DUS4L* 5′ codons were replaced with *OPRL1* 5′ codons confirmed that the identity of the P-site codon is the primary contributor to the difference in readthrough efficiencies between *OPRL1* and *DUS4L* stop signals (Figure 1C).

Although nucleotide biases have been reported 5′ of stop codons (5,6), most studies examining *cis*-acting signals at stop codons focus on 3′ signals. Studying the influence of 5′ *cis*-acting signals on termination efficiency is less straightforward since it is difficult to disentangle the impact of nucleotides in the decoding centre of the ribosome from tRNA features and the influence of amino acids in the vicinity of the peptidyl transferase centre. Few studies have been performed to address the role of 5′ signals in eukaryotes. One study in *Saccharomyces cerevisiae* concluded that both the P-site tRNA and the penultimate amino acid influence decoding of a UGA_C stop signal (63). In another yeast study, Tork *et al.* screened reporter libraries with six randomized nucleotides 5′ of UAG and concluded that neither tRNA identity nor amino acid chemical properties influence termination efficiency (64). Instead they proposed that two 5′ adenines are major determinants of termination efficiency although a recent transcriptome-wide readthrough study in which eRF1 levels were reduced found no such bias (65). One study using a mouse cell line to monitor readthrough, concluded that neither the identity of the terminal amino acid nor the P-site codon frequency affected termination efficiency (66). Instead the nucleotide in the third codon position was shown to be important. This effect could be mediated by the codon nucleotides or by the P-site tRNA.

**Figure 2.**
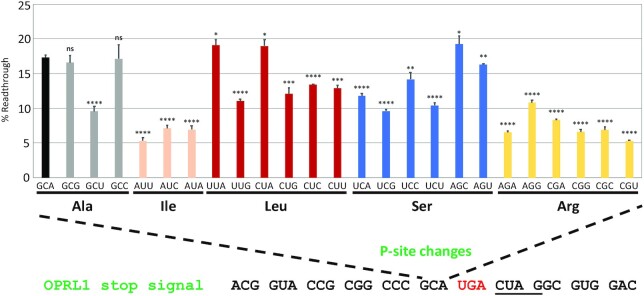
The identity of the P-site codon is a major factor determining *OPRL1* readthrough efficiency. Readthrough efficiencies (% Readthrough) of *OPRL1* stop signals with the indicated P-site codons calculated by dual luciferase assays from HEK293T cells. Statistical significances were determined in comparison with wild-type *OPRL1*. *n* = 3, ns, not significant >0.05; *P <0.05; **P <0.01; ***P <0.001; ****P <0.0001 by Student's two-tailed *t*-test.

To further explore the role of the P-site codon in *OPRL1* readthrough, we systematically tested all alanine and isoleucine codons as well as each of the six-box codons—encoding leucine, serine and arginine (Figure 2). Although there were no significant differences between GCG, GCC and the wild-type GCA alanine codons, GCU in the P-site reduced *OPRL1* readthrough efficiency by almost 50%. With each of the isoleucine codons in the P-site, *OPRL1* readthrough efficiency was reduced to ∼5–7%, with an AUU P-site codon having the lowest efficiency (Figure 2). There are two leucine codons with third position adenines (UUA and CUA), and replacing the *OPRL1* P-site codon with either of these codons had little impact on *OPRL1* readthrough efficiency even though we observed reduced readthrough for each of the remaining four leucine codons (Figure 2). This provides support for the idea that a third position adenine influences termination efficiency. However, this is less clear for arginine codons where both AGA and CGA reporters read through less efficiently than AGG. P-site arginine codons resulted in lower readthrough efficiencies in general compared with most other codons (Figure 2), in agreement with previous observations that positively charged C-terminal amino acids enhance termination efficiency (63). Only serine is decoded by two codons with third position uracils (UCU and AGU), and these P-site codons had varying effects on *OPRL1* readthrough: ∼10% for UCU and ∼16% for AGU (Figure 2). It is noteworthy that in this stop codon context, a P-site serine codon with a third position adenine (UCA) had lower readthrough efficiency than a serine codon with a third position uracil (AGU). Taken together, these data suggest that the effect of the P-site codon on termination cannot be attributed solely to the third position nucleotide or to the encoded amino acid. Nonetheless, the difference in readthrough efficiencies between synonymous codons varied for all five encoded amino acids tested (Figure 2), suggesting that specific features of P-site tRNAs and/or P-site codons influence the efficiency of stop codon readthrough.

### Does the P-site codon influence readthrough efficiency by changing the termination efficiency of the A-site stop codon?

Although termination efficiency and readthrough efficiency are generally assumed to be negatively correlated, an alternative competing process that can take place at stop codons is ribosomal frameshifting. Most known cases of programmed +1 frameshifting take place at locations where ribosomes have reduced ability to decode the A-site codon, i.e. at so-called hungry codons corresponding to rare tRNAs as in the TY1 element (67) or at a stop codon in a weak terminating context (68,69). The identity of the P-site codon when a stop codon is in the A-site has been implicated in both termination efficiency and readthrough (7,63–66) as well as +1 frameshifting (68,70,71), being highly conserved in *prfB* (72), less so in antizymes but still universal in vertebrate antizymes (73). This argues for the importance of the P-site codon for both recoding events. The competition between termination and frameshifting has been studied in the context of known instances of genes whose expression requires +1 ribosomal frameshifting (74–77), where the identity of the P-site codon is believed to be critically important for the efficiency of frameshifting. However, since the magnitude of readthrough or stop codon-dependent +1 frameshifting events may be highly dependent on the efficiencies of their stop signals, then termination and non-termination events must be monitored in parallel to determine those signals important for each event. For example, some of the differences in readthrough efficiencies that we observed between *OPRL1* stop signals with synonymous P-site codons (Figure 2) may be attributed to ribosomal frameshifting, i.e. depending on the specific codon in the P-site, a portion of non-terminating ribosomes may shift into an alternative reading frame. As a result, different stop codon readthrough efficiencies may be reported for stop signals with the same termination efficiencies if some proportion of the non-terminating ribosomes shift into an unmonitored reading frame.

**Figure 3.**
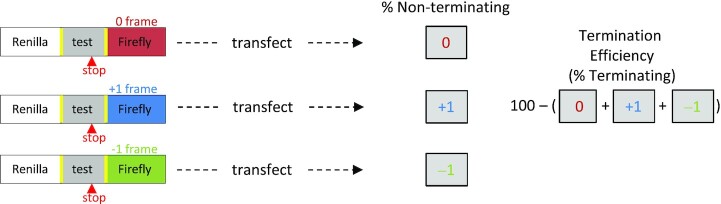
Monitoring translation from all three reading frames enables an estimation of termination efficiency and competition between different non-termination events. Schematic depicting how reporter constructs were designed to measure termination and non-termination events in parallel. Non-termination events are determined by calculating relative luciferase activities (firefly/*Renilla*) from separate test constructs in which firefly luciferase is placed into each of the different reading frames and then dividing by relative luciferase activities from replicate wells of matched in-frame control constructs. Termination efficiencies are calculated by subtracting the sum of the continued translation efficiencies from 100. Yellow rectangles indicate where the StopGo sequences flank the test sequences.

**Figure 4.**
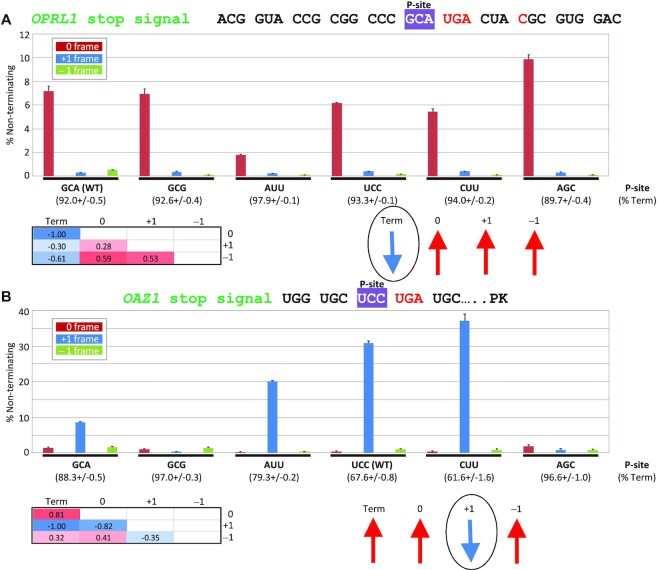
P-site codon identity alters the termination efficiency of the *OPRL1* stop signal and ribosome framing on *OAZ1*. Measurement by dual luciferase reporter assays from HEK293T cells of non-terminating ribosomes during decoding of *OPRL1* stop signals (**A**) or *OAZ1* ORF1 stop signals (**B**) in which their P-site codons were replaced with the indicated codons. Termination efficiencies are shown in parentheses below each P-site codon. Correlations were determined by comparing the means of each of the four competing processes (termination, readthrough and –1/+1 frameshifting) relative to each other and presented as heat maps below each panel. Correlated = red, negatively correlated = blue. *n* = 3. Correlations are also depicted with arrows, and the process that is most affected by the change (negatively correlated with all others) is circled.

To better assess whether different P-site codons impact termination efficiency or the framing of non-terminating ribosomes, we generated separate reporter constructs to monitor translation after the *OPRL1* stop signal from all three reading frames (0, –1 and +1). Since termination and continued translation are mutually exclusive, then termination efficiencies (%) can be calculated by subtracting the sum of the continued elongation events (i.e. 0, –1 and +1 frame translation) from 100 (Figure 3). To permit monitoring of the +1 reading frame, we changed the *OPRL1* UGA_CUAG stop signal to UGA_CUA**C** to prevent +1 (or –2) shifted ribosomes from encountering an almost immediate stop codon. All 0 and –1 frame constructs were also mutated to UGA_CUAC. This single nucleotide change alone reduces readthrough efficiency for *OPRL1* by >50% (Figure 4A compared with Figure 1B).

Monitoring the four competing processes (termination, readthrough and –1/+1 frameshifting) in parallel allows us to determine which process is affected the most in response to changes to the P-site codon. Assuming that changing the P-site codon predominantly affects only a single process, then competing processes should change by compensation and correlate with each other. For example, if termination is slowed, we would expect both readthrough and frameshifting to increase, while if incorporation of a near-cognate tRNA at the stop codon is accelerated we would expect both termination and frameshifting to decrease. In other words, we expect that the most affected process should negatively correlate with the other three, which would correlate with each other.

To apply this principle, we selected P-site codons with varying readthrough efficiencies as determined in Figure 2; however, we excluded GCA reporter data from our correlation analysis because its level of –1 frame translation is highly likely to be due to some level of initiation/reinitiation on a –1 frame AUG. As can be seen from Figure 4A, termination efficiency negatively correlates with all other processes while readthrough efficiency (0 frame) positively correlates with both +1 and –1 translation (see correlation heatmaps in Figure 4A). Thus we conclude that the identity of the P-site codon in the *OPRL1* context predominantly affects termination efficiency, rather than the framing (choice between 0, –1 and + 1 frames) of non-terminating ribosomes.

### P-site codon identity primarily influences +1 frameshifting efficiency at the *OAZ1* ORF1 stop signal

Synthesis of the full-length antizyme from the *OAZ1* mRNA requires highly efficient +1 frameshifting at its ORF1 UCC_UGA stop signal (68). Previous mutagenesis experiments indicated that the P-site codon is instrumental for efficient *OAZ1* frameshifting (68,78). However, these previous studies generally measured frameshifting as the sole alternative to termination, which makes it difficult to conclude whether the P-site codon is important for termination efficiency or frameshifting efficiency. As shown in Figure 4A, by monitoring translation of all three frames, we found that for *OPRL1* the P-site codon does not appear to have a major role in determining which reading frame non-terminating ribosomes continue. If we apply this interpretation to *OAZ1* frameshifting, we would predict that if the P-site codon affects termination efficiency then any change in +1 frameshifting would only be compensatory and all three alternative processes would correlate with each other. To test this prediction, we generated *OAZ1* reporters with the same P-site codons assayed for *OPRL1* (Figure 4A). *OAZ1* frameshifting is stimulated by a highly conserved RNA pseudoknot structure 3′ of the UCC_UGA. To measure translation from all reading frames, we inserted synonymous (with respect to the +1 reading frame) changes to two zero frame stop codons while still maintaining the predicted RNA secondary structure (Supplementary Figure S1A). We observed similar levels of +1 frameshifting between this reporter and a wild-type *OAZ1* reporter (Supplementary Figure S1B).

Correlations between the mean termination efficiencies of all P-site codons and mean efficiencies of continued translation in each reading frame revealed a negative correlation between +1 frameshifting and all other competing processes, which correlate with each other (see correlation heatmaps in Figure 4B). This suggests that in the context of the *OAZ1* ORF1 stop signal, changes to the P-site codon primarily affect +1 frameshifting, while all other events change by compensation.

### Altering nucleotides in the immediate 3′ context of the *OAZ1* ORF1 stop signal impacts different processes

**Figure 5.**
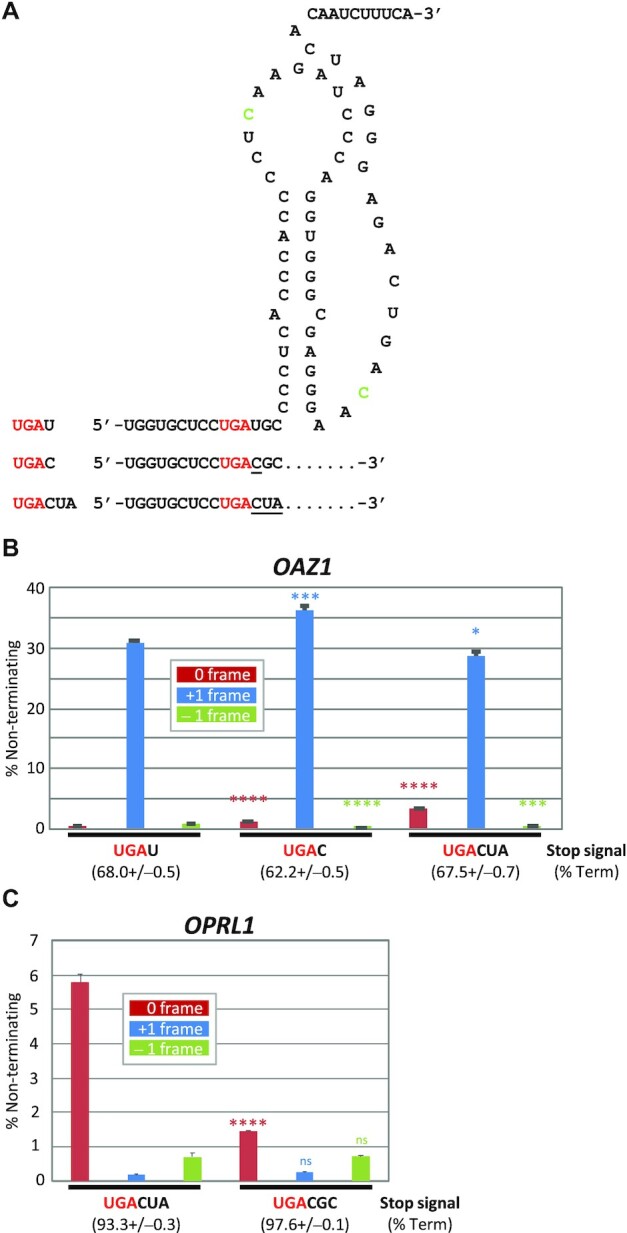
Altering nucleotides in the immediate 3′ context of the *OAZ1* ORF1 stop signal impacts different processes. (**A**) *OAZ1* ORF1 stop context including the modified stimulatory RNA pseudoknot. Changes to the stop codon context are indicated. Nucleotides in green font represent changes to +1 frame stop codons required to allow +1 frame reporter translation (see Supplementary Figure S1A). (**B**) Measurement by dual luciferase reporter assays from HEK293T cells of non-terminating ribosomes during decoding of *OAZ1* ORF1 stop signals with changes to the 3′-adjacent nucleotides indicated. Termination efficiencies are shown in parentheses below each stop signal. Statistical significances were determined in comparison with wild-type *OAZ1* UGA_U. *n* = 3, ns, not significant >0.05; *P <0.05; ***P <0.001; ****P <0.0001 by Student's two-tailed *t*-test. (**C**) Measurement by dual luciferase reporter assays from HEK293T cells of non-terminating ribosomes during decoding of *OPRL1* stop signals with changes to the 3′-adjacent nucleotides indicated. Termination efficiencies are shown in parentheses below each stop signal. ns, not significant >0.05; ****P <0.0001 by Student's two-tailed *t*-test. *n* = 3.

The conservation of U immediately downstream of the UGA codon in *OAZ1* ORF1 is puzzling, since a UGA_C stop signal is a weaker terminator and is expected to promote higher ribosomal frameshifting by compensation. Therefore, we decided to explore how the competition between the main alternative processes is affected by changes in the immediate 3′ context of the *OAZ1* ORF1 stop in the presence of its stimulatory RNA pseudoknot (Figure 5A). As expected *OAZ1* reporters with a UGA_C stop signal have reduced termination efficiency compared with UGA_U, with compensatory changes in both +1 frameshifting and stop codon readthrough (Figure 5B). The drop in the efficiency of –1 frameshifting for the UGA_C stop is most probably due to the elimination of an overlapping AUG codon in the –1 frame which may indicate a low level of reinitiation. Thus, this observation is consistent with the first 3′ nucleotide primarily affecting termination rather than frame selection, although we cannot rule out the possibility that altering the +1 frame codon from GAU to GAC may affect the strength of base pairing by the incoming tRNA. Surprisingly, when we tested UGA_CUA, readthrough levels increased while termination and frameshifting efficiencies decreased compared with the UGA_C context. This suggests that the addition of UA may selectively enhance readthrough rather than weakening termination. In agreement with this, changing the OPRL1 context from UGA_CUA to UGA_CGC resulted in decreased readthrough efficiency, whereas frameshifting levels were not significantly changed (Figure 5C).

### Polyamine levels influence near-cognate decoding of the *OPRL1* stop signal and termination efficiency of the *OAZ1* stop signal

**Figure 6.**
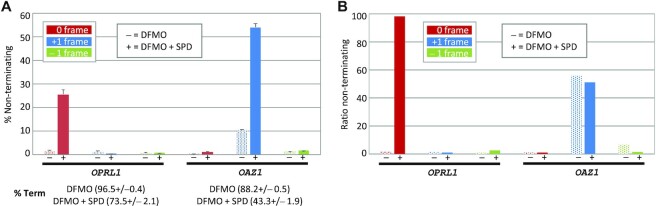
Polyamine levels influence near-cognate decoding of the *OPRL1* stop signal and termination efficiency of the *OAZ1* stop signal. (**A**) Measurement by dual luciferase reporter assays of non-terminating ribosomes during decoding of *OPRL1* and *OAZ1* ORF1 stop signals in HEK293T cells depleted of polyamines (DFMO) or stimulated by 2 mM spermidine (DFMO + SPD). (**B**) Ratios of non-terminating ribosomes from experiments shown in (A). *n* = 3.

Antizyme regulates intracellular polyamine concentrations by destabilizing ornithine decarboxylase, which catalyses the first and rate-limiting step of the polyamine biosynthetic pathway (79). Synthesis of the full-length antizyme from the *OAZ1* mRNA requires +1 frameshifting at its ORF1 UCC_UGA stop signal that is stimulated by polyamine levels (68), forming a complex negative feedback system (80). Stop codon readthrough can also be regulated by polyamine levels (78,81). Whether polyamine levels regulate these recoding events directly, such as by affecting tRNA recognition, or indirectly by altering termination efficiency cannot be determined by measuring only termination or recoding. To address this, we changed intracellular polyamine levels and monitored translation from all reading frames for *OPRL1* and *OAZ1* reporters. Polyamine levels can be depleted by the addition of DFMO, a suicide inhibitor of ornithine decarboxylase. In DFMO-treated cells, termination at the *OPRL1* stop signal is >96% efficient and the non-terminating ribosomes have little framing preference (Figure 6A). However, upon addition of exogenous spermidine, *OPRL1* readthrough was stimulated >20-fold to achieve highly efficient readthrough of >25%. whereas there was negligible translation in either the –1 or the +1 frame. For *OAZ1*, even when polyamines were depleted, termination was still <90% efficient and the majority of non-terminating ribosomes slipped into the +1 reading frame. Upon polyamine addition, termination efficiency reduced to <45% but importantly the ratio of non-terminating ribosomes in each reading frame remained similar (Figure 6B). These experiments suggest that for the *OPRL1* stop signal, increasing the levels of polyamines predominantly acts by enhancing the rate of near-cognate tRNA incorporation at UGA rather than by slowing termination (Figure 6B). In contrast, polyamines predominantly regulate termination efficiency to regulate *OAZ1* +1 frameshifting.

## CONCLUSIONS

When two competing processes are measured, it is difficult to determine which is affected predominantly under a certain condition because a change in one process leads to a reciprocal change in the other. To address this problem, we measured all competing processes that take place at stop codons. In this way, any change in the affected process is expected to lead to reciprocal changes in all other competing processes whose changes correlate. Using this approach, we revealed unexpected facets in how nucleotide and cellular context can influence the decoding of stop codons. Our data suggest that the P-site codon identity predominantly influences the termination efficiency of the *OPRL1* stop codon, whereas it predominantly affects reading frame selection of the non-terminating ribosomes at the *OAZ1* ORF1 stop codon. Interestingly, changes in polyamine levels appear to predominantly influence the rate of near-cognate tRNA incorporation for *OPRL1* readthrough, whereas they influence termination efficiency at *the OAZ1* ORF1 stop codon.

We believe that our approach could be useful for further interrogation of the relationship between individual context elements and understanding how these elements destabilize one of the competing processes to make them particularly sensitive to other factors.

## DATA AVAILABILITY

The exact sequences of oligonucleotides used are available in Supplementary Table S1. Source data for dual luciferase assays are available at DOI: 10.5281/zenodo.7396253.

## Supplementary Material

gkac1180_Supplemental_Files
