# Supplementary material for: Monitoring translation in all reading frames downstream of weak stop codons provides mechanistic insights into the impact of nucleotide and cellular contexts
Source: Nucleic Acids Res. 2022 Dec 19;51(1):304–14. doi: 10.1093/nar/gkac1180 (PMC9841425; doi:10.1093/nar/gkac1180)

**Supplementary Figure 1 A.** Predicted RNA pseudoknot from human *OAZ1* indicating where zero frame stop codons were changed to sense codons. **B.** +1 frameshifting efficiencies of wild-type *OAZ1* and mutant *OAZ1*. N=3.

**Supplementary Table 1** Oligonucleotides used in this study.

Supplementary Figure 1

A

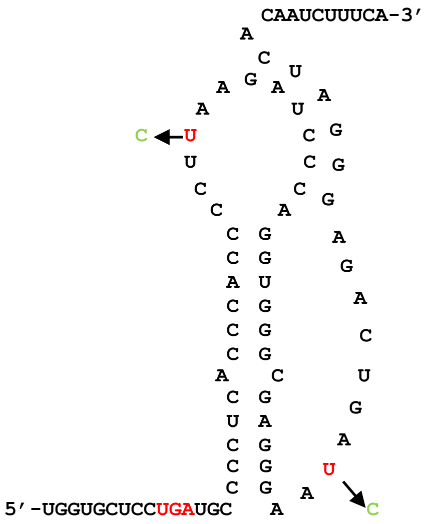


B

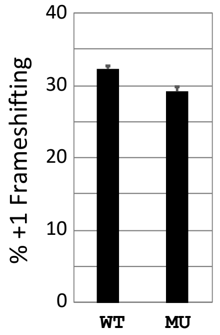

Supplement: gkac1180_Supplemental_Files [file gkac1180_supplemental_files.zip › Supplementary material.pdf]
